# Heteromeric Slick/Slack K^+^ channels show graded sensitivity to cell volume changes

**DOI:** 10.1371/journal.pone.0169914

**Published:** 2017-02-21

**Authors:** Maria A. Tejada, Nadia Hashem, Kirstine Calloe, Dan A. Klaerke

**Affiliations:** Department of Physiology, IKVH, Faculty of Health and Medical Sciences, University of Copenhagen, Dyrlaegevej, Frederiksberg C, Denmark; Tel Aviv University Sackler Faculty of Medicine, ISRAEL

## Abstract

Slick and Slack high-conductance K^+^ channels are found in the CNS, kidneys, pancreas, among other organs, where they play an important role in cell excitability as well as in ion transport processes. They are both activated by Na^+^ and Cl^-^ but show a differential regulation by cell volume changes. Slick has been shown to be regulated by cell volume changes, whereas Slack is insensitive. α-subunits of these channels form homomeric as well as heteromeric channels. It is the aim of this work to explore whether the subunit composition of the Slick/Slack heteromeric channel affects the response to osmotic challenges. In order to provide with the adequate water permeability to the cell membrane of *Xenopus laevis* oocytes, mRNA of aquaporin 1 was co-expressed with homomeric or heteromeric Slick and Slack α-subunits. Oocytes were superfused with hypotonic or hypertonic buffers and changes in currents were measured by two-electrode voltage clamp. This work presents the first heteromeric K^+^ channel with a characteristic graded sensitivity to small and fast changes in cell volume. Our results show that the cell volume sensitivity of Slick/Slack heteromeric channels is dependent on the number of volume sensitive Slick α-subunits in the tetrameric channels, giving rise to graded cell volume sensitivity. Regulation of the subunit composition of a channel may constitute a novel mechanism to determine volume sensitivity of cells.

## Introduction

Slick (Slo2.1) and Slack (Slo2.2) are members of the high-conductance K^+^ channel family, together with BK (Slo1) and Slo3 channels. Only one isoform of Slick channels has been found, however different splice variants of Slack have been described. Slack-A and Slack-B isoforms result in channels which differ in their amino-termini, Slack-A amino-terminus resembles the one of Slick, unlike Slack-B [1]. Slick and Slack form homomeric channels and Slick and Slack-B have been shown to form heteromeric channels [2]. Both of these channels have been primarily studied in the central nervous system (CNS), where they have been suggested to shape the excitability of neurons [3]. However they have also been found in kidneys and pancreas and Slick transcripts were also found in liver, spleen, lung and skeletal muscle [4–6]. Unlike BK and Slo3 channels, Slick and Slack are insensitive to Ca^2+^ but are activated by Na^+^ and Cl^-^. In addition, we have recently shown that both channels are activated by the membrane phospholipid phosphatidylinositol 4,5-bisphosphate (PIP_2_) [7]. Slick and Slack are highly homologous channels, with a 78% sequence identity between them [4], however there are differences in their regulatory mechanisms, such as differential regulation by protein kinase C (PKC) and cell volume changes [8,9].

Cells are often challenged to regulate their volume as they are exposed to a number of physiological processes in relation to e.g sleep/wake cycle, metabolism, salt and water transport, proliferation, migration and apoptosis. Cells can accommodate such changes by a Regulatory Volume Increase (RVI) upon cell shrinkage, or Regulatory Volume Decrease (RVD) upon swelling. During RVD most cells activate K^+^ and Cl^-^ channels, resulting in a release of K^+^ and Cl^-^ together with water [10]. A number of K^+^ channels have been proven to be sensitive to cell volume changes, including KCNQ1, KCNQ4, IK, SK3, Kir4.1/5.1 and Slick channels. On the other hand, channels such as BK, KCNQ2, KCNQ3 and Slack-B are unaffected by changes in cell volume [9,11–13].

Since volume sensitive Slick subunits can form heteromeric channels with volume insensitive Slack-B subunits, we hypothesized that the relative contribution of Slick and Slack subunits could affect the volume sensitivity of heteromeric channels. Our results clearly indicate that when Slick and Slack-B subunits co-associate to form heteromeric channels, they show a characteristic cell volume sensitivity that is intermediate between the strong cell volume sensitivity of homomeric Slick channels and the insensitivity of homomeric Slack-B channels. In addition, the number of volume sensitive Slick α-subunits in the tetrameric channel complex determines the degree of volume sensitivity of the heteromeric channel.

## Methods

### Molecular biology

To generate concatemeric Slick/Slick, Slack/Slack and Slick/Slack channels, we used an uracil excision-based cloning method (USER cloning) [14] on cDNA coding for Slick and Slack channels cloned into pOX vector, kindly provided by Dr. L. Salkoff. We used for this study the Slack-B isoform due to its ability to form heteromeric channels with Slick, and it will be further referred in this paper as Slack. Briefly, a high-fidelity PCR was performed with Pfu Turbo C_x_ polymerase (Stratagene) with uracil-containing primers, in order to generate uracil overhangs, necessary for the junction of the C-terminus of Slick with the N-terminus of Slack. At the same time, uracil overhangs were created in order to facilitate the introduction of the concatemeric fragment into pXOOMu vector, containing a USER cloning cassette (Table 1).

**Table 1:** PCR primers for generating Slick/Slick, Slack/Slack and Slick/Slack concatemeric channels.

Concatemer	Primer Name	Sequence
	U-SlickN-Fw	5’–*GGC TTA* AU ATG GTT GAT TTG GAG AGC GAA G– 3’
**Slick/Slick**	U-SlickC-Rv	5’–*GGT TTA A***U** TCA AAG TTG AGT TTC CTC CCG– 3’
	U-SlickJ-Fw	5’–ACT CAA CTT A**U**G GTT GAT TTG GAG AGC– 3’
	U_SlickJ_Rv	5’–AT AAG TTG AG**U** TTC CTC CCG AGA ATC TTG ACC– 3’
	U-SlackN_Fw	*5’–GGC TTA A***U** ATG GCG CGG GCC AAG*– 3’*
**Slack/Slack**	U-SlackC_Rv	*5’–GGT TTA A***U** TCA GAG CTG GGT CTC ATC CCG*– 3**’*
	U-SlackJ_Fw	5’–GAG ACC CAG CTC A**U**G GCG CGG– 3’
	U-SlackJ_Rv	5’–AT GAG CTG GG**U** CTC ATC CCG GGT CTC– 3’
	U-SlickN-Fw	5’–*GGC TTA A***U** ATG GTT GAT TTG GAG AGC GAA G– 3’
**Slick/Slack**	U-SlackC-Rv	5’–*GGT TTA A***U**T CAG AGC TGG GTC TCA TCC CG– 3’
	U-SlickJ-Rv	5’–AT AAG TTG AG**U** TTC CTC CCG AGA ATC TTG ACC– 3’
	U-SlackJ-Fw	5’–ACT CAA CTT A**U**G GCG CGG GCC AAG CTG– 3’

Oligo-DNA primers were designed for the junction of 2 Slick, 2 Slack or Slick/Slack cDNA sequences. Primer names noted with “N” and “C” contain 5’ extensions (upstream uracil) that complement the overhangs of the pXOOMu cloning vector (sequences of overhangs are in italics). Primers designed to join the C-terminus of one channel with the N-terminus of following channel are noted with “J” in their names. Bold and underlined residues are uracils necessary for the excision-based cloning method.

The PCR products were later treated with USER enzyme mix (NEB), containing an uracil DNA glycosylase and a DNA-glycosylase-lyase. The concomitant action of this enzyme mix excised the uracil and ligated both subunits together and the concatemeric fragment to the pXOOMu vector. Positive clones were verified by PCR of single colonies, followed by sequencing at Eurofins MWG Operon (Ebersberg, Germany).

Slick/Slick, Slack/Slack and Slick/Slack concatemers were cloned, as previously described into pXOOMu vector. Aquaporin 1 (AQP1) in pBluescript was a courtesy of Dr. P. Agre. Plasmid DNA was linearized with *NotI* for monomeric Slick and Slack, *XhoI* for concatemeric Slick/Slick, Slack/Slack and Slick/Slack constructs and *PstI* for AQP1 (New England Biolabs, Ipswich, MA, USA). Linearized plasmid DNA was purified using the High Pure PCR Purification Kit (Roche, Mannheim, Germany) and was *in-vitro* transcribed with the mMESSAGE mMACHINE kit from Ambion (Austin, Texas, USA). Messenger RNA (mRNA) was purified with the MEGAclear kit (Ambion) according to manufacturer’s instructions.

### Ethics statement

*Xenopus laevis* frogs were purchased from Nasco (Fort Atkinson) and were housed in glass tanks (Acqua Schwarz Stand-alone V-60 system, Göttingen, Germany) according to animal welfare. Tanks were filled with filtered and UV-sterilized water, which was daily monitored for pH, conductivity and a temperature of 19°C. The frogs were housed in groups of similar size and gender and were fed twice a week with floating frog food. Oocytes were harvested surgically from frogs and all efforts were made in order to minimize animal suffering. The procedure to remove oocytes was conducted under tricaine anesthesia (2 g L^-1^) and frogs were left to recover in a separate tank with a slope in order to facilitate breathing by having the animal’s nostrils above the water level. Frogs were frequently monitored until conscious and were returned to their original tanks the following day. Animals and their surgical incisions were regularly inspected for signs of infection on the following days after surgery. Surgical oocyte harvest was performed once a year on each frog for up to 8 years. *Xenopus laevis* frogs were euthanized after this period or in cases of strong bleeding during surgery or wound opening after surgery. The method of euthanasia used in this study was sedation by tricaine until loss of consciousness, followed by decapitation and removal of vital organs (brain). This procedure was specifically approved and carried out in strict accordance with the guidelines of The Danish National Animal Experiments Inspectorate [15].

### Heterologous expression in *Xenopus laevis* oocytes

Oocytes were prepared as previously described by Grunnet *et al* [11]. 10 ng of mRNA mixture of homomeric Slick and AQP1 (3:1 ratio, respectively) were injected (in 50 nl) into oocytes. Same amount and ratio were used for injections of homomeric Slack and AQP1. For co-expression of concatemeric Slick/Slack mRNA with monomeric Slick or Slack mRNA, the ratio expected to produce channels made of 3 subunits of one type and 1 different subunit was injected, in a total of 10 ng/oocyte. Co-injections of the Slick/Slack concatemer with either Slick/Slick or Slack/Slack mRNA were in 1:1 ratio. Oocytes were stored at 19°C in Kulori medium (90 mM NaCl, 1 mM KCl, 1 mM MgCl_2_, 1 mM CaCl_2_, 5 mM HEPES, pH 7.4).

### Electrophysiology and data analysis

Currents were measured by two-electrode voltage clamp (TEVC), 3–5 days post-injection, using an OC-275B amplifier (Warner Instruments, Hamden, Connecticut, USA). Electrodes were pulled using a Micropipette Puller P-97 (Sutter Instruments, Novato, California, USA) and filled with 1 M KCl. Electrode resistance was 0.5–1.5 MΩ. Two voltage clamp protocols were used: A Step Protocol consisting of 500 ms steps ranging from -100 to +80 mV in 20 mV increments from a holding potential of -80 mV, and with an interpulse interval of 4 seconds; a Pulse Protocol consisting of a step to +80 mV for 500 ms, from a holding potential of -80 mV, with an interpulse interval of 3 seconds.

Volume changes were induced by superfusion with hypotonic media (0 mM D-mannitol, 137 mOsm kg^-1^) or hypertonic media (100 mM D-mannitol, 239 mOsm kg^-1^) from isotonic media (50 mM D-mannitol, 188 mOsm kg^-1^). All these media also contained 65 mM NaCl, 1 mM KCl, 1 mM MgCl_2_, 1 mM CaCl_2_ and 5 mM HEPES, pH 7.4. D-mannitol and HEPES were from Sigma, other chemicals were from Merck.

Data acquisition and analysis were performed using pClamp 10.4 (Molecular Devices, Sunnyvale, CA, USA) and GraphPad Prism®. Data are presented as mean ± SEM. Statistical differences were assessed by paired Student’s *t*-tests, one-way ANOVA with Tukey’s post-test; or two-way ANOVA for grouped analysis with Bonferroni post-tests. Statistical significance of *p*-values: * (p<0.05); ** (p<0.005); *** (p<0.0005).

## Results

### Co-expression of Slick and Slack and their volume sensitivity

**Fig 1:**
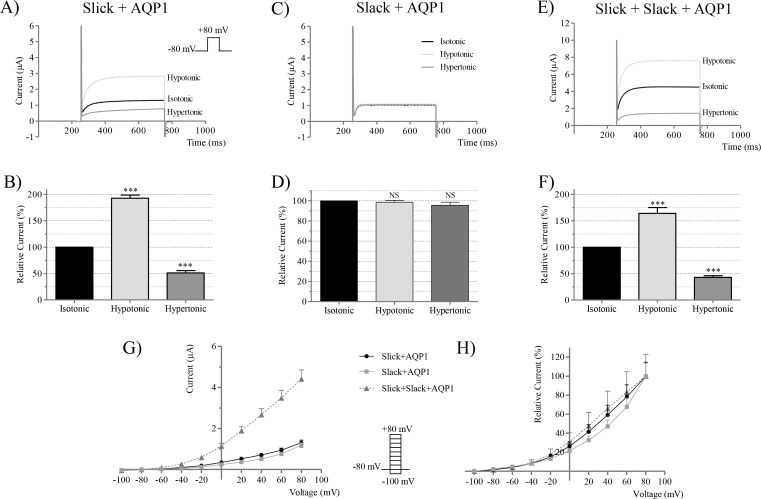
Volume regulation of Slick and Slack homomeric and heteromeric channels. Co-expression of Slick, Slack and AQP1 in *Xenopus laevis* oocytes. Currents were stimulated by a pulse protocol (inset in A). Representative currents at + 80 mV (A, C, E), as well as maximal currents normalized to isotonic buffers (B, D, F) are shown for oocytes exposed from isotonic (black) to hypotonic (light grey) and hypertonic buffers (dark grey). (A, B) Slick+AQP1. (C, D) Slack+AQP1. (E, F) Slick+Slack+AQP1. (G) Current–Voltage relationship for oocytes expressing homomeric Slick or Slack channels and oocytes co-expressing both subunits together with AQP1. Currents were stimulated by a step protocol (inset) and were measured at the end of the depolarizing steps. (H) Normalized current-voltage relationship for Slick+AQP1, Slack+AQP1 and Slick+Slack+AQP1 (*n* = 4–10).

The volume sensitivity of homomeric Slick and Slack channels (Fig 1A, 1B, 1C and 1D) was evaluated by co-expression with AQP1 in *Xenopus laevis* oocytes to facilitate fast changes in cell volume upon changes in tonicity. Currents were recorded by TEVC. In agreement with our previous findings [9], homomeric Slick channels were highly sensitive to changes in cell volume (Fig 1A and 1B), whereas homomeric Slack channels were insensitive to volume changes (Fig 1C and 1D). The degree of volume sensitivity of Slick channels was evaluated in every experiment, due to a slight sensitivity variation between batches of oocytes. Slick and Slack α-subunits were co-expressed resulting in heteromeric channels. Slick/Slack heteromeric currents were activated by cell swelling, increasing currents to 164 ± 11% (*n* = 8) and inhibited by cell shrinkage to 43 ± 3% (*n* = 8) of control (Fig 1E and 1F). Interestingly, the volume response of the Slick/Slack heteromeric channels was intermediate between the highly volume sensitive homomeric Slick channels (Fig 1A and 1B) and the insensitive homomeric Slack channels (Fig 1C and 1D). In agreement with Chen *et al* [2], the heteromeric channels expressed significantly higher currents compared to homomeric Slick or Slack channels (Fig 1G). To determine the voltage-sensitivity of the channels, the normalized current/voltage (I/V) relationship was calculated (Fig 1H). Similarly to homomeric Slick and Slack channels, the Slick/Slack heteromeric channels displayed only a minor voltage dependency and were not significantly different from the homomeric channels.

### Slick/Slack concatemeric channels are volume sensitive

**Fig 2:**
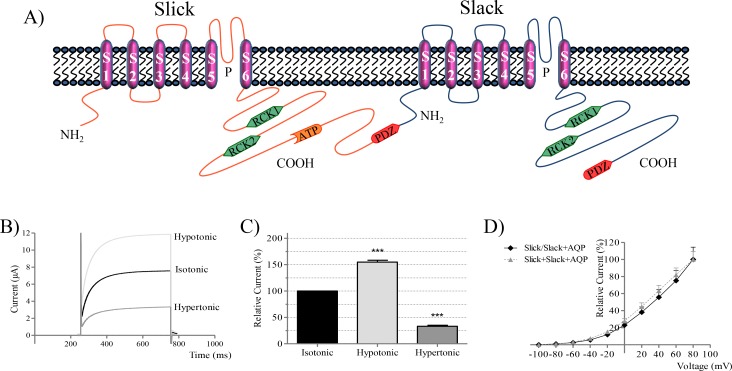
Slick/Slack concatemeric channels. A Slick/Slack concatemeric subunit was constructed by the junction of the C-terminus of the Slick channel sequence with the N-terminus of Slack as represented in (A) and expressed in *Xenopus laevis* oocytes. (B) Representative heteromeric Slick/Slack currents recorded during osmotic challenges, upon stimulation by a pulse protocol as in Fig 1. (C) Summarized data of the effect of osmotic challenges on heteromeric channels. (D). I/V curves normalized to the maximal current for oocytes expressing the Slick/Slack concatemer or co-expressing homomeric Slick and Slack together with AQP1 (*n* = 10).

To determine the subunit composition of Slick/Slack heteromeric channels in relation to their volume sensitivity, we constructed a concatemeric construct by joining the C-terminus of Slick with the N-terminus of Slack (Fig 2A). Slick/Slack concatemeric channels were successfully expressed in *Xenopus laevis* oocytes and produced large currents with slow activation kinetics similarly to non-concatemeric Slick/Slack channels (cf. Fig 1). Furthermore, Slick/Slack concatemeric channels were also significantly activated by hypotonic buffers and currents increased to 155 ± 3% (*n* = 6). In contrast, currents decreased in hypertonic buffers to 33 ± 2% (*n* = 6) of control (Fig 2B and 2C). Similarly to our co-expression experiments, the volume sensitivity of Slick/Slack concatemeric channels was smaller compared to that of homomeric Slick channels but higher compared to that of the volume insensitive Slack channel (cf. Fig 1). The I/V relationship of Slick/Slack concatemeric channels was similar to that of the channels formed by co-expression of homomeric Slick and Slack α-subunits (Fig 2D).

### Graded cell volume sensitivity of Slick/Slack heteromeric channels in different configurations

**Fig 3:**
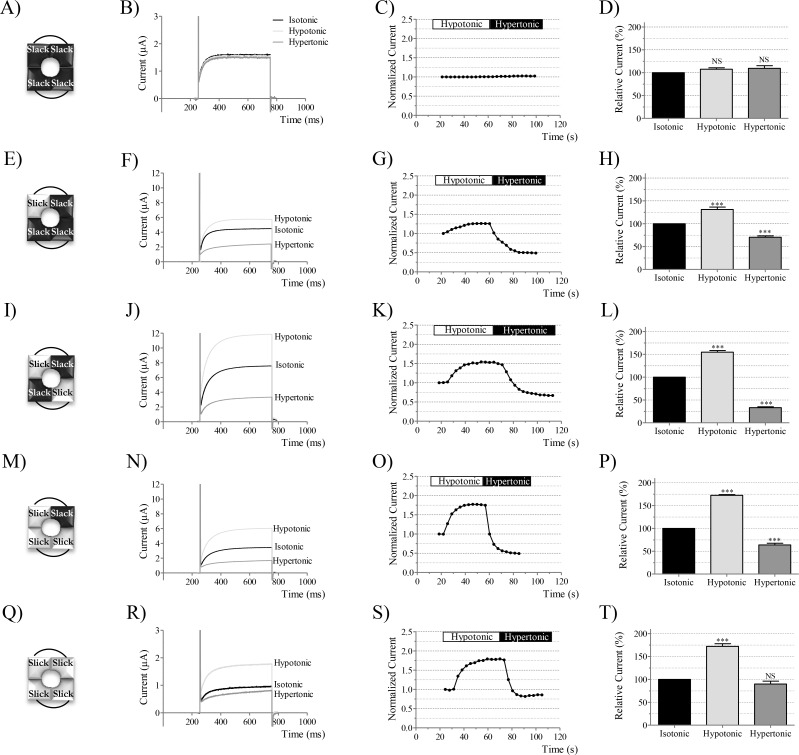
Volume sensitivity of homomeric and heteromeric Slick/Slack channels in different configurations. In (A), Slack homodimers were synthesized and expressed in *Xenopus laevis* oocytes. (B) Maximal currents at the end of a depolarizing step to +80 mV for a representative oocyte expressing Slack/Slack, during cell volume challenges. Currents were stimulated by a pulse protocol as in Fig 1. (C) Representative maximal currents over time for a Slack/Slack expressing oocyte exposed to osmotic challenges. The time and period of application of hypotonic and hypertonic buffers are indicated by white and black boxes, respectively. Currents were normalized to isotonic conditions. (D) Maximal currents at the end of the +80 mV step for Slack/Slack expressing oocytes upon volume changes (*n* = 17). Currents are shown relative to isotonic buffers. (E) Heteromeric Slick/Slack channels formed by 3 Slack and 1 Slick subunits and the volume sensitivity of a representative oocyte (F, G) and summarized data (*n* = 6) (H). In (I), heteromeric channels were formed by 2 Slick and 2 Slack subunits and their sensitivity to cell volume is represented in (J), (K) and (L) (*n* = 6). (M) Heteromeric channels made by 3 Slick and 1 Slack subunit and their response osmotic challenges for a representative recording in (N, O), and for 10 oocytes in (P). In (Q), Slick homodimers were constructed and expressed in oocytes. (R) and (S) show current changes upon changes in cell volume for representative oocytes, or for 9 oocytes (T).

Since Slick/Slack heteromeric channels in 2:2 conformation showed intermediate volume sensitivity compared to Slick and Slack channels alone, we set out to investigate the volume response of heteromeric channels in 3:1 conformation. Therefore, we constructed concatemeric Slack channels by joining two Slack subunits in tandem (Fig 3A) and concatemeric Slick channels in a similar manner (Fig 3Q). We verified that Slick/Slick and Slack/Slack concatemeric channels behaved as channels formed by single α-subunits. Slack concatemeric channels were insensitive to changes in cell volume (Fig 3B–3D), whereas expression of Slick concatemers resulted in volume sensitive channels, with currents activated to 172 ± 6% (*n* = 9) in the hypotonic buffers, and with a tendency towards reduced currents upon cell shrinkage (Fig 3R–3T). Hypertonic currents were not statistically different from currents recorded under isotonic buffers (Fig 3T).

Next, we co- expressed concatemeric Slick/Slack and concatemeric Slack/Slack mRNA in a 1:1 ratio, resulting in heteromeric channels consisting of 3 Slack and 1 Slick subunits (Fig 3E). The resulting heteromeric channel was volume sensitive (Fig 3F) and currents were activated by cell swelling to 131 ± 5% (*n* = 6) and inhibited by cell shrinkage to 70 ± 3% (*n* = 6) of control (Fig 3G and 3H). The volume sensitivity of these heteromeric channels formed by 3 Slack and 1 Slick subunits was smaller compared to heteromeric channels formed by equal number of Slick and Slack subunits (Fig 3I–3L). We then explored the volume sensitivity of heteromeric channels formed by 3 Slick and 1 Slack α-subunit, by co-expression of the Slick/Slack concatemer together with the Slick/Slick concatemer (1:1 ratio) in oocytes (Fig 3M). The resulting channels were also volume sensitive (Fig 3N) and currents were activated by hypotonic buffers to 172 ± 1% (*n* = 10) and inhibited by hypertonic buffers to 64 ± 4% (*n* = 10) compared to controls (Fig 3O and 3P).

All results were confirmed by co-expression of Slick/Slack concatemers with Slick or Slack monomeric α-subunits and clearly highlight the translation efficiency of *Xenopus laevis* oocytes as a heterologous expression system (S1 Fig).

## Discussion

We have recently shown that Slick channels are highly sensitive to cell volume changes, unlike Slack channels [9]. In the present study, we show for the first time, that Slick/Slack heteromeric channels respond to volume changes in a graded manner, according to the number of volume sensitive α-subunits in the tetrameric channel complex.

### Properties of heteromeric Slick/Slack channels

**Fig 4:**
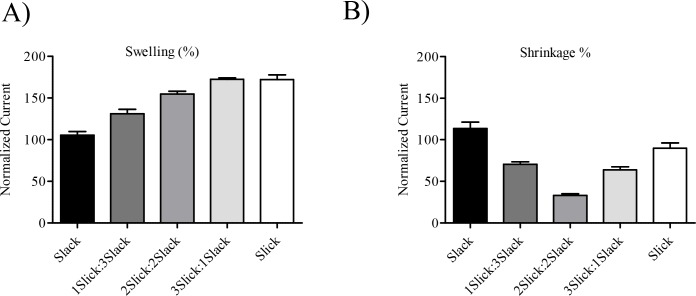
Swelling and shrinking response of homomeric and heteromeric Slick/Slack channels. Maximal currents measured at the end of +80 mV and normalized to isotonic buffers for homomeric Slack, and Slick, as well as heteromeric Slick/Slack in different configurations (Slick 1:3 Slack; Slick 2:2 Slack; Slick 3:1 Slack) in hypotonic (A) and hypertonic (B) buffers (*n* = 6–10)

In agreement with Chen *et al* [2], we found that expression of heteromeric channels resulted in higher current amplitudes compared to homomeric channels. To explore the functional properties, including the volume sensitivity of heteromeric channels in different configurations, we performed a series of concatemeric constructs. The properties of homodimeric channels were not different from the properties of channels consisting of monomeric α-subunits. Intrinsic channel properties, such as voltage dependence, activation kinetics, macroscopic currents and volume sensitivity were similar in oocytes expressing Slack monomers or Slack/Slack homodimers. Slick channels formed by monomeric α-subunits as well as homodimers, also showed similar intrinsic channel properties; however, small changes in the volume sensitivity of Slick/Slick concatemeric channels could be observed. The formation of Slick channels, by the association of Slick/Slick concatemers, produced channels that were strongly activated by cell swelling, as channels formed by monomeric Slick α-subunits. However, cell shrinkage did not significantly reduce Slick/Slick concatemeric current (Fig 4). The background for this phenomenon is still unclear, and it may be related to the concatenation process, as currents recorded from un-concatenated Slick are reduced by shrinkage. In addition, our results reveal that Slick/Slack heteromeric channels, resulting from the association of concatemers, also recapitulated the high currents and slow activation kinetics of Slick/Slack heteromers formed by monomeric Slick and Slack subunits.

### Cell volume regulation of Slick/Slack heteromeric channels

Modulation of heteromeric channels by cell volume has been studied for a number of K^+^ channels [11,13,16,17]. Recently, the pore forming subunits of the volume regulated anion channel (VRAC) were identified and it was found that the functional VRAC channel is made from the association of different subunits in a heteromeric form [17]. Slick and Slack are found both as homomeric as well as heteromeric channels in the CNS [2,18–20]. Despite being homologous, these channels differ in their cell volume regulation. The present study investigated the response to cell volume changes of heteromeric channels formed by highly volume sensitive Slick α-subunits in various combinations with volume insensitive Slack α-subunits. We found that heteromeric channels formed by 2 Slick and 2 Slack subunits respond to small changes in cell volume with a sensitivity that is intermediate in between homomeric Slick and Slack channels. Heteromeric channels consisting of 3 Slick and 1 Slack α-subunit respond to cell swelling with higher sensitivity than channels made from equal numbers of Slick and Slack subunits, yet their sensitivity was slightly lower than homomeric Slick channels. In addition, heteromeric channels formed by 3 Slack and 1 Slick α- subunits responded to cell swelling with a sensitivity that was intermediate between heteromers formed by 2 Slick and 2 Slack subunits but significantly different from the insensitive homomeric Slack channels (Fig 4). This clearly demonstrates that the number of volume sensitive Slick α-subunits in the tetrameric Slick/Slack channel complex determines the response of the channel to cell volume changes. The presence of a single volume sensitive subunit into a tetrameric channel is enough to confer volume sensitivity. To our knowledge, this is the first time it has been demonstrated that the number of volume sensitive α-subunits in the tetrameric channel determines the degree of volume sensitivity of the resulting heteromeric channel.

### Physiological and pathophysiological implications

Slick and Slack homomeric channels are widely distributed in the CNS and possibly in the heart [3,20]. In addition, co-localization of both of these channels was found in many neurons of rat and mouse brain, including olfactory bulb and the Medial Nucleus of the Trapezoid Body (MNTB) [2,20]. Slick and Slack may play a role shaping the excitability of neurons by the modulation of hyperpolarization [3,21,22]. Due to their volume sensitivity, Slick/Slack heteromeric channels may be important for the regulation of water intake in neurons, however their contribution to volume regulation *in vivo* remains to be determined. Moreover, homomeric channels were suggested to play an important role under episodes of hypoxia/ischemia, which is normally accompanied by cell swelling and elevated intracellular Na^+^ concentrations due to failure in the Na^+^/K^+^ ATP_ase_ [23]. Both, cell swelling and elevated Na^+^ concentrations may result in activation of Slick/Slack heteromeric channels. This would in turn hyperpolarize the membrane potential, reducing excitability and protecting cells from damage and degeneration caused by depolarizations and excitotoxicity under ischemic conditions, similarly to other members of the Slick and Slack channel family, namely the Ca^2+^-activated BK, SK and IK channels [24]. This makes Slick and Slack channels attractive candidates for a protective role under episodes of ischemia. Interestingly, despite the K_ATP_ channels have been shown to play a hyperpolarizing role during early stages of ischemia, these channels close again during later phases, yet K^+^ efflux occurs via alternative, not clearly identified channels [25]. Slick and Slack have been found co-localized in neurons of the hippocampus (CA1), in a similar manner as K_ATP_ channels [2]. Given the different association of Slick and Slack subunits, it is a possibility that the different conformations of volume sensitive heteromers could better accommodate and protect the cells against later stages of an ischemic injury, when the activity of other protective K^+^ channels has stopped or as a safety net during the different ischemic phases.

### Conclusions

We have clearly demonstrated that the number of volume sensitive α-subunits in the tetrameric channel determines the degree of volume sensitivity of the resulting heteromeric channel. Thus, regulation of the subunit composition of a channel may constitute a novel mechanism to determine volume sensitivity of cells.

## Supporting information

S1 Fig Volume sensitivity of Slick/Slack heteromeric channels formed by concatemeric and monomeric subunits. Schematic representation of the formation of Slick/Slack heteromeric channels by the co-expression of Slick/Slack concatemeric channel together monomeric Slick subunits (A) or with monomeric Slack subunits (D) in *Xenopus laevis* oocytes. Currents were stimulated with a pulse protocol as in Fig 1. (B) Representative currents at +80 mV for concatemeric Slick/Slack expressed with monomeric Slick in response to osmotic challenges and in (C) summarized data, normalized to isotonic values, for 7 oocytes. (E) Maximal currents for concatemeric Slick/Slack expressed with monomeric Slack in response to osmotic challenges and (F) summarized data, *n* = 10.
(TIF)
