# Supplementary figures and images for: Heteromeric Slick/Slack K+ channels show graded sensitivity to cell volume changes
Source: PLoS One. 2017 Feb 21;12(2):e0169914. doi: 10.1371/journal.pone.0169914 (PMC5319697; doi:10.1371/journal.pone.0169914)

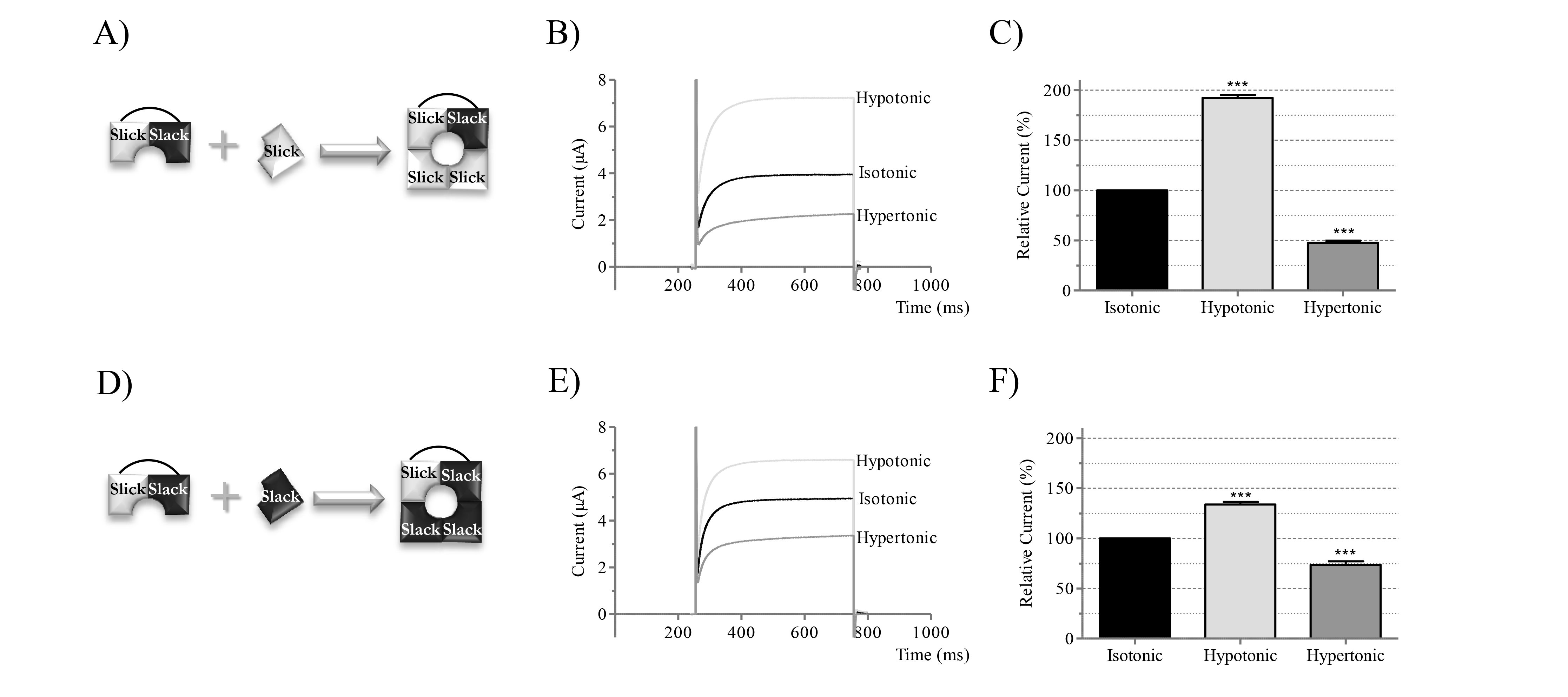

Supplement: S1 Fig — Schematic representation of the formation of Slick/Slack heteromeric channels by the co-expression of Slick/Slack concatemeric channel together monomeric Slick subunits (A) or with monomeric Slack subunits (D) in Xenopus laevis oocytes. Currents were stimulated with a pulse protocol as in Fig 1. (B) Representative currents at +80 mV for concatemeric Slick/Slack expressed with monomeric Slick in response to osmotic challenges and in (C) summarized data, normalized to isotonic values, for 7 oocytes. (E) Maximal currents for concatemeric Slick/Slack expressed with monomeric Slack in response to osmotic challenges and (F) summarized data, n = 10. (TIF) [file pone.0169914.s001.tif]
